# Psychological distress, fear and coping among Malaysians during the COVID-19 pandemic

**DOI:** 10.1371/journal.pone.0257304

**Published:** 2021-09-10

**Authors:** Ahmed Suparno Bahar Moni, Shalimar Abdullah, Mohammad Farris Iman Leong Bin Abdullah, Mohammed Shahjahan Kabir, Sheikh M. Alif, Farhana Sultana, Masudus Salehin, Sheikh Mohammed Shariful Islam, Wendy Cross, Muhammad Aziz Rahman

**Affiliations:** 1 Advanced Medical and Dental Institute, Universiti Sains Malaysia, Bertam, Kepala Batas, Penang, Malaysia; 2 Faculty of Medicine, Pusat Perubatan Universiti Kebangsaan Malaysia, Kuala Lumpur, Malaysia; 3 Faculty of Medicine, Quest International University, Ipoh, Perak, Malaysia; 4 School of Public Health and Preventive Medicine, Monash University, Melbourne, Victoria, Australia; 5 Telstra Health, Melbourne, Victoria, Australia; 6 Melbourne School of Population and Global Health, University of Melbourne, Carlton, Victoria, Australia; 7 School of Health, Federation University Australia, Berwick, Victoria, Australia; 8 Institute for Physical Activity and Nutrition, Deakin University, Burwood, Victoria, Australia; 9 Australian Institute for Primary Care and Ageing, La Trobe University, Melbourne, Victoria, Australia; 10 Department of Noncommunicable Diseases, Bangladesh University of Health Sciences (BUHS), Dhaka, Bangladesh; 11 Faculty of Public Health, Universitas Airlangga, Surabaya, Indonesia; Università degli Studi di Firenze: Universita degli Studi di Firenze, ITALY

## Abstract

**Introduction:**

The COVID-19 pandemic has enormously affected the psychological well-being, social and working life of millions of people across the world. This study aimed to investigate the psychological distress, fear and coping strategies as a result of the COVID-19 pandemic and its associated factors among Malaysian residents.

**Methods:**

Participants were invited to an online cross-sectional survey from Aug-Sep 2020. The study assessed psychological distress using the Kessler Psychological Distress Scale, level of fear using the Fear of COVID-19 Scale, and coping strategies using the Brief Resilient Coping Scale. Univariate and multivariate logistic regression analyses were conducted to adjust for potential confounders.

**Results:**

The mean age (±SD) of the participants (N = 720) was 31.7 (±11.5) years, and most of them were females (67.1%). Half of the participants had an income source, while 216 (30%) identified themselves as frontline health or essential service workers. People whose financial situation was impacted due to COVID-19 (AOR 2.16, 95% CIs 1.54–3.03), people who drank alcohol in the last four weeks (3.43, 1.45–8.10), people who were a patient (2.02, 1.39–2.93), and had higher levels of fear of COVID-19 (2.55, 1.70–3.80) were more likely to have higher levels of psychological distress. Participants who self-isolated due to exposure to COVID-19 (3.12, 1.04–9.32) and who had moderate to very high levels of psychological distress (2.56, 1.71–3.83) had higher levels of fear. Participants who provided care to a family member/patient with a suspected case of COVID-19 were more likely to be moderately to highly resilient compared to those who did not.

**Conclusion:**

Vulnerable groups of individuals such as patients and those impacted financially during COVID-19 should be supported for their mental wellbeing. Behavioural interventions should be targeted to reduce the impact of alcohol drinking during such crisis period.

## Introduction

The world is currently facing a pandemic due to the rapid spread of coronavirus disease 2019 (COVID-19), caused by severe acute respiratory syndrome coronavirus 2 (SARS-CoV-2). As of 23^rd^ March 2021, an estimated 124,291,475 confirmed cases and around 2,735,205 deaths have been attributed to COVID-19 affecting more than 219 countries and territories across the world [1]. Malaysia has reported 334,156 confirmed cases with 1,238 cumulative deaths with a case fatality rate of 0.4% [1]. Although the case fatality rate was low in Malaysia compared to other developed countries like USA or UK, people were anxious as the virus could spread rapidly from one person to another through direct or indirect contact [2].

In Malaysia, the first COVID-19 case was detected on 25 January 2020 [3]. With a surge in cases thereafter, physical distancing rules, restrictions on social gatherings, appropriate use of face masks, Movement Control Order (MCO), Conditional Movement Control Order (CMCO), extended movement control order, and border closures were implemented by the Malaysian Government between mid-March to August-2020 to curb the spread of the disease [4]. However, Malaysia has seen a resurgence of COVID-19 cases and is currently facing a third wave of infection and is under the second CMCO from 9-November-2020 in all states except Perlis, Kelantan, Pahang and Sarawak. Malaysia has launched COVID-19 vaccination program on the 24^th^ February 2021 [5]. The impact of all these spatial distancing policies and the uncertainty of returning to normalcy have direct and indirect impact on social life as well as mental wellbeing of the community people. Those interim actions such as MCOs or lockdowns, physical distancing and quarantine have reportedly led to heightened fears, stress and anxiety amongst individuals globally.

A recent review found women, younger individuals, those living in rural areas, those with lower socioeconomic status, those at higher-risk of COVID-19 infection and longer media exposure to be associated with higher levels of anxiety and depression [6]. Individual studies have shown that the COVID-19 pandemic affected people in different countries in different ways with some groups being more vulnerable than others. In Australia, pre-existing mental health conditions, increased smoking and alcohol during the lockdown and high levels of fear and being female were associated with higher levels of psychological distress [7]. Similarly, in the UK, females, younger age, lower annual income, smokers and co-morbidity were associated with poor mental health [8]. While in Italy, female gender, negative affect and detachment were associated with higher levels of stress [9]. In some studies in China, frequent and prolonged social media exposure during the COVID-19 pandemic was found to be strongly associated with anxiety and depression [10].

Previous studies have reported the negative influence of pandemics on psychological well-being, which can lead to acute depression and anxiety [7, 11]. Evidence suggests that frontline healthcare workers, who were directly involved in the collection of samples, diagnosis, treatment, and care of patients during an outbreak were at higher risk of developing psychological distress and mental health symptoms [12]. Previous evidence documented immediate psychological impacts amongst frontline healthcare workers with symptoms of anxiety, distress, depression, fear of spreading infection to family, friends and colleagues [7, 13]. Lower sleep quality due to anxiety and stress, which eventually reduced self-efficacy exponentially among the medical staff has also been reported [14].

Only recently studies have emerged to show the negative impact of the pandemic on children, older people, pregnant women, university students, people with weight issues and the general population as a whole. An Iranian study revealed effect of fear of COVID-19 was significantly associated with depression, anxiety, suicidal intention and mental quality of life among the pregnant women [15]. In a study with older people, fear of COVID-19 significantly mediated the association between perceived health status, and insomnia, mental health and COVID-19 preventative behaviours [16]. A recent study among university students from Indonesia, Taiwan and Thailand found that Thai students had the highest level of anxiety but limited resources to fight the COVID-19 pandemic, whereas Taiwanese students were more negatively affected by information gathering from the internet; such less perceived satisfactory support was associated with more suicidal thoughts among Indonesian students [17]. Stressors of COIVD-19 pandemic could also result in behaviour impairments of children and adolescents, which could potentially impact psychological wellbeing in early life and adulthood [18].

Studies on the impact of COVID-19 on mental health are limited in Malaysia and most of them were conducted amongst students. In one study using online survey, out of 983 Malaysian students, 20.4%, 6.6%, and 2.8% experienced minimal to moderate, marked to severe, and most extreme levels of anxiety, respectively. Female gender, age under 18 years, pre-university level of education, management studies, and staying alone were significantly associated with higher levels of anxiety. The main stressors included financial constraints, remote online teaching, and uncertainty about the future regarding study and career also affecting the mental health [2]. In another study amongst Malaysian university students, the prevalence of anxiety was much higher; 30.5% were experiencing mild, 31.1% moderate, and 26.1% severe anxiety; age >20 years, Chinese ethnicity, decreased family income, co-morbid conditions, and spending time watching COVID-related news and infected friends and relatives were found to be associated with increased anxiety [19]. In another study in Malaysia age <25 years and females were more likely to have higher levels of fear of COVID-19; however, 70% of the respondents were also students in this study [20].

There is limited evidence regarding the impact of COVID-19 on psychological distress, fear and coping strategies as a whole and amongst community members and healthcare workers in Malaysia. We, therefore, conducted this study to understand the extent of the mental health burden in the community settings in Malaysia during the COVID-19 pandemic. The study will identify population subgroups more at risk of developing poor mental health outcomes and enable policy makers to guide resource planning and design psychosocial interventions targeted to these high-risk and vulnerable groups of population.

## Materials and methods

### Study design and settings

A cross-sectional study was conducted between August and September 2020. An online survey link was shared in different online platforms, including Facebook, Twitter and LinkedIn inviting online users to participate in this study.

### Study population

Study participants included patients, university students and healthcare professionals residing in Malaysia. To be eligible, participants had to be 18 years or above and were literate enough to respond to an online questionnaire in English. The participants who took <1 minute to complete the questionnaire, were excluded during analyses.

### Sampling

Sample size was calculated using OpenEpi [21]. Considering 32.6 million population of Malaysia [22], 30% estimated prevalence of stress amongst Malaysians [23, 24], at 95% confidence intervals and 80% power, the estimated minimum sample size was 323. Snowball sampling technique was used to recruit the study participants. Once any participant filled up the online questionnaire, h/she forwarded the survey link to own personal/professional networks.

### Data collection

Google form was used to develop the study questionnaire. The first page included participant information statement and the consent form. Participants, who provided consents, could move to the next screen. There were two screening questions to determine eligibility of the study participants, one was age and the other was location of residence. Eligible participants accessed the full study questionnaire and responses were collected anonymously. The online survey link was shared through university/hospital staff/students’ emails, text messages, WhatsApp and other social media platforms such as Facebook, Twitter and LinkedIn. Patients visiting any healthcare settings or university students within the defined study period were informed about the study and of the online link by the respective healthcare professionals or university faculty members.

### Study tool

We used the same survey questionnaire (except residence location/region in Malaysia) which was used earlier by the Australian investigators included in this study [7]. Three validated tools were included in the survey questionnaire. The Kessler Psychological Distress Scale (K10) tool having ten items was used to assess psychological distress [25], the Fear of COVID-19 scale (FCV-19S) having seven items was used to assess the levels of fear [26], and the Brief Resilient Coping Scale (BRCS) having four items was used to assess the levels of coping [27]. Each of those tools collected responses using a 5-point likert scale and the scoring was categorised as discussed in earlier study [7]. Reliability of using these tools had also been examined in a recent study [28]. The questionnaire was pre-tested and no changes were made.

### Data analyses

Data from Google forms were downloaded and analysed using STATA v.12. Continuous variables were described using descriptive statistics such as mean standard deviations, and proportions. Scoring in the K10 scale was re-defined into low (score 10–15) and moderate to very high (score 16–50), the FCV-19S scale to low (score 7–21) and high (score 22–35) and BRCS scale categorised into low (score 4–13) and medium to high (score 14–20) resilient coping. We used univariate and multivariate logistic regression to investigate the associations. The multivariate models were adjusted for socio-demographic variables such as age, gender, living status, country of birth, education, and employment status.

### Ethics

Ethics approval was obtained from the Human Research Ethics Committee (HREC) at Universiti Sains Malaysia (USM/JEPeM/COVID19-40). Data were collected anonymously and could not be linked back to identify any participant. Contact details of Befrienders was included at the end of the online questionnaire, allowing participant/s to access necessary support in case of distress during filling questionnaire.

## Results

A total of 720 individuals participated in this study. Mean age (±SD) of the participants was 31.7 (±11.5) years, and most of them (56.7%) were in the age group 18–29 years. More than two-thirds of the participants (67.1%) were females. A quarter of the study population (27.1%) was from Penang, and another quarter (22.9%) was from Perak in Malaysia. Almost all of them (90.8%) were born in Malaysia. A third of the study population (30%) identified themselves as frontline or essential service workers, and a third (31.9%) was identified as patients. Details of the characteristics of the study population are presented in Table 1.

**Table 1:** Characteristics of the study population.

Characteristics	Total, n(%)
Total study participants	720
**Age (in years)**	**702**
Mean (±SD)	31.7 (11.5)
Range	19 to 76
**Age groups**	**702**
18–29 years	398 (56.7)
30–59 years	282 (40.2)
≥60 years	22 (3.1)
**Gender**	**720**
Male	235 (32.6)
Female	483 (67.1)
Others	2 (0.3)
**Location in Malaysia**	**720**
Johor	24 (3.3)
Kedah	50 (6.9)
Kelantan	60 (8.3)
Kuala Lumpur	72 (10.0)
Kuala Terengganu	5 (0.7)
Malacca	7 (1.0)
Nigari Sembilan	8 (1.1)
Pahang	16 (2.2)
Penang	195 (27.1)
Perak	165 (22.9)
Perlis	3 (0.4)
Sabah	13 (1.8)
Sarawak	6 (0.8)
Selangor	96 (13.3)
**Living status**	**718**
Live without family members (on your own/shared house/others)	136 (18.9)
Live with family members (partner and/or children)	559 (77.9)
**Born in Malaysia**	**720**
No	66 (9.2)
Yes	654 (90.8)
**Completed level of education**	**716**
Secondary	113 (15.8)
Diploma	119 (16.6)
Degree (Bachelor)	301 (42.0)
Masters and above	183 (25.6)
**Current employment condition**	**710**
Unemployed/Home duties (No source of income)	309 (43.5)
Jobs affected by COVID-19 (lost job/working hours reduced/afraid of job loss)	46 (6.5)
Have an income source (employed/Government benefits)	355 (50.0)
**Perceived distress due to change of employment status**	**699**
A little to none	482 (69.0)
Moderate to a great deal	217 (31.0)
**Self-identification as a frontline or essential service worker**	**720**
No	504 (70.0)
Yes	216 (30.0)
**COVID-19 impacted financial situation**	**720**
No	379 (52.6)
Yes	341 (47.4)
**Co-morbidities**	**612**
No	478 (78.1)
Psychiatric/Mental health problem	20 (3.3)
Other co-morbidities*	114 (18.6)
**Smoking**	**720**
Never smoker	656 (91.1)
Ever smoker (Daily/Non-daily/Ex)	64 (8.9)
**Current alcohol drinking (last 4 weeks)**	**713**
No	665 (93.3)
Yes	48 (6.7)
**Increased alcohol drinking over the last 4 weeks**	**48**
No	34 (70.8)
Yes	14 (29.2)
**Provided care to a family member/patient with known/suspected case of COVID-19**	**715**
No	647 (90.5)
Yes	68 (9.5)
**Experience related to COVID-19 pandemic (multiple responses possible)**	**688**
No known exposure to COVID-19	638 (92.7)
I had recent overseas travel history and was in self-quarantine	10 (1.5)
I have been tested negative for COVID-19 but self-isolating	40 (5.8)
**Self-identification as a patient (visited a healthcare provider in the last 4 weeks)**	**715**
No	487 (68.1)
Yes	228 (31.9)
**Healthcare service use in the last 4 weeks**	**301**
Telehealth consultation/Use of national helpline	240 (79.7)
In-person visit to a healthcare provider	45 (15.0)
Used both services	16 (5.3)
**Healthcare service use to overcome COVID-19 related stress in the last 4 weeks**	**707**
No	689 (97.5)
Yes	18 (2.5)

* Stroke/Hypertension/Hyperlipidaemia/Diabetes/Cancer/Chronic respiratory illness

About two-thirds of the study participants (62.1%) experienced moderate to very high levels of psychological distress. Only a quarter (27.1%) reported high levels of fear of COVID-19 and two-thirds of the participants (65.1%) were identified as having medium to high resilient coping (Tables 2–4).

**Table 2:** Level of psychological distress among the study participants.

Anxiety and Depression Checklist (K10) (last 4 weeks)	Total, n(%)
**About how often did you feel tired out for no good reason?**	**720**
None	184 (25.6)
A little	182 (25.3)
Sometime	248 (34.4)
Most of the time	83 (11.5)
All the time	23 (3.2)
**About how often did you feel nervous?**	**720**
None	229 (31.8)
A little	221 (30.7)
Sometime	206 (28.6)
Most of the time	55 (7.6)
All the time	9 (1.3)
**About how often did you feel so nervous that nothing could calm you down?**	**720**
None	378 (52.5)
A little	178 (24.7)
Sometime	129 (17.9)
Most of the time	31 (4.3)
All the time	4 (0.6)
**About how often did you feel hopeless?**	**720**
None	361 (50.1)
A little	176 (24.4)
Sometime	120 (16.7)
Most of the time	51 (7.1)
All the time	12 (1.7)
**About how often did you feel restless or fidgety?**	**720**
None	296 (41.1)
A little	200 (27.8)
Sometime	164 (22.8)
Most of the time	46 (6.4)
All the time	14 (1.9)
**About how often did you feel so restless you could not sit still?**	**720**
None	382 (53.1)
A little	175 (24.3)
Sometime	134 (18.6)
Most of the time	22 (3.1)
All the time	7 (1.0)
**About how often did you feel so depressed?**	**720**
None	310 (43.1)
A little	205 (28.5)
Sometime	139 (19.3)
Most of the time	48 (6.7)
All the time	18 (2.5)
**About how often did you feel that everything was an effort?**	**720**
None	194 (26.9)
A little	224 (31.1)
Sometime	178 (24.7)
Most of the time	98 (13.6)
All the time	26 (3.6)
**About how often did you feel so sad that nothing could cheer you up?**	**720**
None	325 (45.1)
A little	194 (26.9)
Sometime	144 (20.0)
Most of the time	42 (5.8)
All the time	15 (2.1)
**About how often did you feel worthless?**	**720**
None	378 (52.5)
A little	178 (24.7)
Sometime	107 (14.9)
Most of the time	34 (4.7)
All the time	23 (3.2)
**K10 score (total)**	**720**
Mean (±SD)	20.0 (8.3)
Range	10 to 50
**Level of psychological distress (K10 categories)**	**720**
Low (score 10–15)	273 (37.9)
Moderate (score 16–21)	177 (24.6)
High (score 22–29)	151 (21.0)
Very high (score 30–50)	119 (16.5)

**Table 3:** Level of fear of COVID-19 among the study participants.

Fear of COVID-19 Scale (FCV-19S) individual items	Total, n(%)
**I am most afraid of COVID-19**	**720**
Strongly disagree	106 (14.7)
Somewhat disagree	109 (15.1)
Neither agree nor disagree	150 (20.8)
Somewhat agree	237 (32.9)
Strongly agree	118 (16.4)
**It makes me uncomfortable to think about COVID-19**	**720**
Strongly disagree	136 (18.9)
Somewhat disagree	118 (16.4)
Neither agree nor disagree	165 (22.9)
Somewhat agree	230 (31.9)
Strongly agree	71 (9.9)
**My hands become clammy when I think about COVID-19**	**720**
Strongly disagree	333 (46.3)
Somewhat disagree	166 (23.1)
Neither agree nor disagree	164 (22.8)
Somewhat agree	42 (5.8)
Strongly agree	15 (2.1)
**I am afraid of losing my life because of COVID-19**	**720**
Strongly disagree	188 (26.1)
Somewhat disagree	113 (15.7)
Neither agree nor disagree	154 (21.4)
Somewhat agree	180 (25.0)
Strongly agree	85 (11.8)
**When watching news and stories about COVID-19 on social media, I become nervous or anxious**	**720**
Strongly disagree	165 (22.9)
Somewhat disagree	139 (19.3)
Neither agree nor disagree	161 (22.4)
Somewhat agree	216 (30.0)
Strongly agree	39 (5.4)
**I cannot sleep because I’m worrying about getting COVID-19**	**720**
Strongly disagree	413 (57.4)
Somewhat disagree	135 (18.8)
Neither agree nor disagree	129 (17.9)
Somewhat agree	33 (4.6)
Strongly agree	10 (1.4)
**My heart races or palpitates when I think about getting COVID-19**	**720**
Strongly disagree	346 (48.1)
Somewhat disagree	123 (17.1)
Neither agree nor disagree	142 (19.7)
Somewhat agree	90 (12.5)
Strongly agree	19 (2.6)
**FCV-19S score (total)**	**720**
Mean (±SD)	17.5 (6.3)
Range	7 to 35
**Level of fear of COVID-19 (FCV-19S categories)**	**720**
Low (score 7–21)	525 (72.9)
High (score 22–35)	195 (27.1)

**Table 4:** Coping during COVID-19 pandemic among the study participants.

Brief Resilient Coping Scale (BRCS) individual items	Total, n(%)
**I look for creative ways to alter difficult situations**	**720**
Does not describe me at all	24 (3.3)
Does not describe me	55 (7.6)
Neutral	289 (40.1)
Describes me	270 (37.5)
Describes me very well	82 (11.4)
**Regardless of what happens to me, I believe I can control my reaction to it**	**720**
Does not describe me at all	7 (1.0)
Does not describe me	56 (7.8)
Neutral	249 (34.6)
Describes me	302 (41.9)
Describes me very well	106 (14.7)
**I believe I can grow in positive ways by dealing with difficult situations**	**720**
Does not describe me at all	10 (1.4)
Does not describe me	33 (4.6)
Neutral	182 (25.3)
Describes me	348 (48.3)
Describes me very well	147 (20.4)
**I actively look for ways to replace the losses I encounter in life**	**720**
Does not describe me at all	26 (3.6)
Does not describe me	40 (5.6)
Neutral	281 (39.0)
Describes me	304 (42.2)
Describes me very well	69 (9.6)
**BRCS score (total)**	**720**
Mean (±SD)	14.4 (2.7)
Range	4 to 20
**Level of coping (BRCS categories)**	**720**
Low resilient coping (score 4–13)	251 (34.9)
Medium resilient coping (score 14–16)	345 (47.9)
High resilient coping (score 17–20)	124 (17.2)

Table 5 shows the univariate and multivariate analyses regarding factors associated with psychological distress. Moderate to very high levels of psychological distress was associated with impacted financial situation due to COVID-19 (AOR 2.16, 95% CIs 1.54–3.03, p<0.001), alcohol drinking in the last four weeks (AOR 3.43, 95% CIs 1.45–8.10, p<0.01), being a patient (AOR 2.02, 95% CIs 1.39–2.93, p<0.001), and having higher levels of fear of COVID-19 (AOR 2.55, 95% CIs 1.70–3.80, p<0.001). However, those in the older age groups i.e. 30–59 years (AOR 0.51, 95% CIs 0.27–0.95, p<0.05), those of ≥60 years old (AOR 0.07, 95% CIs 0.01–0.37, p<0.01) and those who had medium to highly resilient coping (AOR 0.54, 95% CIs 0.38–0.77, p<0.01) were less likely experience higher psychological distress (Table 5).

**Table 5:** Factors associated with high psychological distress among the study population (based on K10 scoring).

Characteristics	Moderate to Very High (score 16–50), n(%)	Low (score 10–15), n(%)	Unadjusted analyses			Adjusted analyses		
			p	OR	95% CIs	p	AOR	95% CIs
Total study participants	447	273	** **		** **			
**Age groups**	**433**	**269**	** **		** **			
18–29 years	278 (64.2)	120 (44.6)		1			1	
30–59 years	153 (35.3)	129 (48.0)	***<0*.*001***	***0*.*51***	***0*.*37–0*.*70***	***0*.*035***	***0*.*51***	***0*.*27–0*.*95***
≥60years	2 (0.5)	20 (7.4)	***<0*.*001***	***0*.*04***	***0*.*01–0*.*19***	***0*.*002***	***0*.*07***	***0*.*01–0*.*37***
**Gender**	**445**	**273**	** **		** **			
Male	126 (28.3)	109 (39.9)		1			1	
Female	319 (71.17)	164 (60.1)	***0*.*001***	***1*.*68***	***1*.*22–2*.*31***	0.186	1.57	0.80–3.07
**Living status**	**426**	**269**	** **		** **			
Live without family members (on your own/shared house/others)	90 (21.1)	46 (17.1)		1			1	
Live with family members (partner and/or children)	336 (78.9)	223 (82.9)	0.193	0.77	0.52–1.14	0.729	0.80	0.23–2.79
**Born in Malaysia**	**447**	**273**	** **		** **			
No	53 (11.9)	13 (4.8)		1			1	
Yes	394 (88.1)	260 (95.2)	***0*.*001***	***0*.*37***	***0*.*20–0*.*70***	0.257	0.60	0.24–1.46
**Completed level of education**	**444**	**272**	*** ***		*** ***			
Secondary	76 (17.1)	37 (13.6)	** **	1	** **		1	
Diploma	76 (17.1)	43 (15.8)	0.587	0.86	0.50–1.48	0.744	0.78	0.17–3.54
Degree (Bachelor)	209 (47.1)	92 (33.8)	0.670	1.11	0.70–1.76	0.466	0.52	0.09–2.99
Masters and above	83 (18.7)	100 (36.8)	***<0*.*001***	***0*.*40***	***0*.*25–0*.*66***	0.466	0.66	0.22–2.01
**Current employment condition**	**441**	**269**	** **		** **			
Unemployed/Home duties	167 (37.9)	142 (52.8)		1			1	
Jobs affected by COVID-19 (lost job/working hours reduced/afraid of job loss)	29 (6.6)	17 (6.3)	0.254	1.45	0.77–2.75	0.698	1.24	0.42–3.69
Have an income source (employed/Government benefits)	245 (55.6)	110 (40.9)	***<0*.*001***	***1*.*89***	***1*.*38–2*.*60***	0.887	1.08	0.36–3.27
**Perceived distress due to change of employment status**	**434**	**265**	** **		** **			
A little to none	319 (73.5)	163 (61.5)	** **	1	** **		1	
Moderate to a great deal	115 (26.5)	102 (38.5)	***0*.*001***	***0*.*58***	***0*.*42–0*.*80***	0.981	NA	NA
**Self-identification as a frontline or essential service worker**	**447**	**273**	** **		** **			
No	289 (64.7)	215 (78.8)		1			1	
Yes	158 (35.3)	58 (21.2)	***<0*.*001***	***2*.*03***	***1*.*43–2*.*87***	0.071	1.59	0.96–2.63
**COVID-19 impacted financial situation**	**447**	**273**	** **		** **			
No	200 (44.7)	179 (65.6)		1			1	
Yes	247 (55.3)	94 (34.4)	***<0*.*001***	***2*.*35***	***1*.*72–3*.*21***	***0*.*000***	***2*.*16***	***1*.*54–3*.*03***
**Co-morbidities**	**372**	**240**						
No	274 (73.7)	204 (85.0)		1			1	
Psychiatric/Mental health problem	10 (2.7)	10 (4.2)	0.518	0.74	0.30–1.82	0.254	0.24	0.02–2.79
Other co-morbidities*	88 (23.7)	26 (10.8)	***<0*.*001***	***2*.*52***	***1*.*57–4*.*05***	0.285	1.47	0.73–2.97
**Smoking**	**447**	**273**	** **		** **			
Never smoker	405 (90.6)	251 (91.9)		1			1	
Ever smoker (Daily/Non-daily/Ex)	42 (9.4)	22 (8.1)	0.541	1.18	0.69–2.03	0.734	0.83	0.28–2.47
**Current alcohol drinking (last 4 weeks)**	**443**	**270**	** **		** **			
No	403 (91.0)	262 (97.0)		1			1	
Yes	40 (9.0)	8 (3.0)	***0*.*002***	***3*.*25***	***1*.*50–7*.*06***	***0*.*005***	***3*.*43***	***1*.*45–8*.*10***
**Provided care to a family member/patient with known/suspected case of COVID-19**	**443**	**272**	** **		** **			
No	394 (88.9)	253 (93.0)		1			1	
Yes	49 (11.1)	19 (7.0)	0.071	1.66	0.95–2.88	0.120	1.60	0.89–2.89
**Experience related to COVID-19 pandemic**	**423**	**265**	** **		** **			
No known exposure to COVID-19	385 (91.0)	253 (95.5)	** **	1			1	
I had recent overseas travel history and was in self-quarantine	7 (1.7)	3 (1.1)	0.538	1.53	0.39–5.98	NA	NA	NA
I have been tested negative for COVID-19 but self-isolating	31 7.3)	9 (3.4)	***0*.*035***	***2*.*26***	***1*.*06–4*.*83***	0.592	0.74	0.25–2.23
**Self-identification as a patient (visited a healthcare provider in the last 4 weeks)**	**444**	**271**	*** ***		*** ***			
No	280 (63.1)	207 (76.4)	*** ***	1	*** ***		1	
Yes	164 (36.9)	64 (23.6)	***<0*.*001***	***1*.*89***	***1*.*35–2*.*66***	***0*.*000***	***2*.*02***	***1*.*39–2*.*93***
**Healthcare service use in the last 4 weeks**	**207**	**94**	** **		** **			
Telehealth consultation/Use of national helpline	34 (16.4)	11 (11.7)		1			1	
In-person visit to a healthcare provider	160 (77.3)	80 (85.1)	0.243	0.65	0.31–1.34	0.473	0.76	0.35–1.62
Used both services	13 (6.3)	3 (3.2)	0.643	1.40	0.34–5.84	0.551	1.57	0.36–6.84
**Level of fear of COVID-19 (FCV-19S categories)**	**447**	**273**	** **		** **			
Low (score 7–21)	301 (67.3)	224 (82.1)		1			1	
High (score 22–35)	146 (32.7)	49 (17.9)	***<0*.*001***	***2*.*22***	***1*.*54–3*.*20***	***0*.*000***	***2*.*55***	***1*.*70–3*.*80***
**Level of coping (BRCS categories)**	**447**	**273**	** **		** **			
Low resilient coping (score 4–13)	176 (39.4)	75 (27.5)		1			1	
Medium to high resilient coping (score 14–20)	271 (60.6)	198 (72.5)	***0*.*001***	***0*.*58***	***0*.*42–0*.*81***	***0*.*001***	***0*.*54***	***0*.*38–0*.*77***
**Healthcare service use to overcome COVID-19 related stress in the last 4 weeks**	**440**	**267**	** **		** **			
No	426 (96.8)	263 (98.5)		1			1	
Yes	14 (3.2)	4 (1.5)	0.168	2.16	0.70–6.63	0.196	2.13	0.68–6.69

Adjusted for: age, gender, living status, born in Malaysia, education and employment

* Cardiac diseases/Stroke/Hypertension/Hyperlipidemia/Diabetes/Cancer/Chronic respiratory disease

Table 6 shows the univariate and multivariate analyses regarding factors associated with fear of COVID-19. Study participants who had been tested negative for COVID-19 but were self-isolating (AOR 3.12, 95% CIs 1.04–9.32, p<0.05) and those who had moderate to very high levels of psychological distress (AOR 2.56, 95% CIs 1.71–3.83, p<0.001) also had high levels of fear. Conversely, study participants who were born in Malaysia (AOR 0.39, 95% CIs 0.18–0.86, p<0.05) and who drank alcohol in the last four weeks (AOR 0.26, 95% CIs 0.10–0.68, p<0.01) had lower levels of fear in this study (Table 6).

**Table 6:** Factors associated with high levels of fear of COVID-19 among the study population (based on FCV-19S scoring).

Characteristics	High (score 22–35), n(%)	Low (score 7–21), n(%)	Unadjusted analyses			Adjusted analyses		
			p	OR	95% CIs	p	AOR	95% CIs
Total study participants	195	525	** **		** **			
**Age groups**	**191**	**511**	** **		** **			
18–29 years	105 (55.0)	293 (57.3)		1			1	
30–59 years	81 (42.4)	201 (39.3)	0.500	1.12	0.80–1.58	0.493	1.25	0.66–2.39
≥60 years	5 (2.6)	17 (3.3)	0.705	0.82	0.30–2.28	0.599	0.70	0.18–2.67
**Gender**	**195**	**523**	** **		** **			
Male	65 (33.3)	170 (32.5)		1			1	
Female	130 (66.7)	353 (67.5)	0.833	0.96	0.68–1.37	0.795	1.10	0.54–2.26
**Living status**	**187**	**508**	** **		** **			
Live without family members (on your own/shared house/others)	38 (20.3)	98 (19.3)		1			1	
Live with family members (partner and/or children)	149 (79.7)	410 (80.7)	0.762	0.94	0.62–1.43	0.276	2.53	0.48–13.5
**Born in Malaysia**	**195**	**525**	** **		** **			
No	25 (12.8)	41 (7.8)		1			1	
Yes	170 (87.2)	484 (92.2)	***0*.*038***	***0*.*58***	***0*.*34–0*.*98***	***0*.*020***	***0*.*39***	***0*.*18–0*.*86***
**Completed level of education**	**194**	**522**	*** ***		*** ***			
Secondary	35 (18.0)	78 (14.9)	** **	1	** **		1	
Diploma	29 (14.9)	90 (17.2)	0.262	0.72	0.40–1.28	0.239	0.32	0.05–2.14
Degree (Bachelor)	81 (41.8)	220 (42.1)	0.413	0.82	0.51–1.32	0.251	0.29	0.04–2.38
Masters and above	49 (25.3)	134 (25.7)	0.437	0.81	0.49–1.37	0.152	0.32	0.07–1.52
**Current employment condition**	**96**	**259**	** **		** **			
Unemployed/Home duties	88 (45.8)	221 (42.7)		1			1	
Jobs affected by COVID-19 (lost job/working hours reduced/afraid of job loss)	8 (4.2)	38 (7.3)	0.119	0.53	0.24–1.18	0.212	0.46	0.13–1.56
Have an income source (employed/Government benefits)	96 (50.0)	259 (50.0)	0.680	0.93	0.66–1.31	0.893	0.92	0.29–2.98
**Perceived distress due to change of employment status**	**189**	**510**	** **		** **			
A little to none	130 (68.8)	352 (69.0)	** **	1	** **		1	
Moderate to a great deal	59 (31.2)	158 (31.0)	0.952	1.01	0.71–1.45	NA	NA	NA
**Self-identification as a frontline or essential service worker**	**195**	**525**	** **		** **			
No	138 (70.8)	366 (69.7)		1			1	
Yes	57 (29.2)	159 (30.3)	0.784	0.95	0.66–1.36	0.112	0.64	0.37–1.11
**COVID-19 impacted financial situation**	**195**	**525**	** **		** **			
No	92 (47.2)	287 (54.7)		1			1	
Yes	103 (52.8)	238 (45.3)	0.074	1.35	0.97–1.88	0.119	1.33	0.93–1.89
**Co-morbidities**	**161**	**451**						
No	123 (76.4)	355 (78.7)		1			1	
Psychiatric/Mental health problem	3 (1.9)	17 (3.8)	0.288	0.51	0.15–1.77	0.648	0.50	0.03–9.77
Other co-morbidities*	35 (21.7)	79 (17.5)	0.282	1.28	0.82–2.00	0.585	0.81	0.37–1.75
**Smoking**	**195**	**525**	** **		** **			
Never smoker	172 (88.2)	484 (92.2)		1			1	
Ever smoker (Daily/Non-daily/Ex)	23 (11.8)	41 (7.8)	0.095	1.58	0.92–2.71	0.413	1.61	0.52–4.99
**Current alcohol drinking (last 4 weeks)**	**193**	**520**	** **		** **			
No	186 (96.4)	479 (92.1)		1			1	
Yes	7 (3.6)	41 (7.9)	***0*.*044***	***0*.*44***	***0*.*19–0*.*99***	***0*.*006***	***0*.*26***	***0*.*10–0*.*68***
**Provided care to a family member/patient with known/suspected case of COVID-19**	**194**	**521**	** **		** **			
No	175 (90.2)	472 (90.6)		1			1	
Yes	19 (9.8)	49 (9.4)	0.875	1.05	0.60–1.83	0.60	1.17	0.65–2.11
**Experience related to COVID-19 pandemic**	**183**	**505**	** **		** **			
No known exposure to COVID-19	164 (89.6)	474 (93.9)	** **	1			1	
I had recent overseas travel history and was in self-quarantine	4 (2.2)	6 (1.2)	0.314	1.93	0.54–6.91	NA	NA	NA
I have been tested negative for COVID-19 but self-isolating	15 (8.2)	25 (5.0)	0.104	1.73	0.89–3.37	***0*.*042***	***3*.*12***	***1*.*04–9*.*32***
**Self-identification as a patient (visited a healthcare provider in the last 4 weeks)**	**194**	**521**	*** ***		*** ***			
No	132 (68.0)	355 (68.1)	*** ***	1	*** ***		1	
Yes	62 (32.0)	166 (31.9)	0.980	1.00	0.71–1.43	0.924	1.02	0.70–1.48
**Healthcare service use in the last 4 weeks**	**79**	**222**	** **		** **			
Telehealth consultation/Use of national helpline	12 (15.2)	33 (14.9)		1			1	
In-person visit to a healthcare provider	61 (77.2)	179 (80.6)	0.860	0.94	0.46–1.93	0.413	0.73	0.34–1.57
Used both services	6 (7.6)	10 (4.5)	0.417	1.65	0.49–5.53	0.804	1.18	0.33–4.24
**Level of psychological distress (K10 categories)**	**195**	**525**	** **		** **			
Low (score 10–15)	49 (25.1)	224 (42.7)		1			1	
Moderate to Very High (score 16–50)	146 (74.9)	301 (57.3)	***<0*.*001***	***2*.*22***	***1*.*54–3*.*20***	***<0*.*001***	***2*.*56***	***1*.*71–3*.*83***
**Level of coping (BRCS categories)**	**195**	**525**	** **		** **			
Low resilient coping (score 4–13)	77 (39.5)	174 (33.1)		1			1	
Medium to high resilient coping (score 14–20)	118 (60.5)	351 (66.9)	0.112	0.76	0.54–1.07	0.074	0.72	0.50–1.03
**Healthcare service use to overcome COVID-19 related stress in the last 4 weeks**	**191**	**516**	** **		** **			
No	185 (96.9)	504 (97.7)		1			1	
Yes	6 (3.1)	12 (2.3)	0.541	1.36	0.50–3.68	0.453	1.47	0.54–4.02

Adjusted for: age, gender, living status, born in Malaysia, education and employment

* Cardiac diseases/Stroke/Hypertension/Hyperlipidemia/Diabetes/Cancer/Chronic respiratory disease

Study participants who provided care to a family member/patient with known/suspected case of COVID-19 had medium to high resilient coping (AOR 1.87, 95% CIs 1.01–3.46, p<0.05), whereas participants with moderate to very high levels of psychological distress had low resilient coping (AOR 0.54, 95% CIs 0.38–0.76, p<0.01) (Table 7).

**Table 7:** Factors associated with coping among the study population (based on BRCS scoring).

Characteristics	Medium to High (score 14–20), n(%)	Low (score 4–13), n(%)	Unadjusted analyses			Adjusted analyses		
			p	OR	95% CIs	p	AOR	95% CIs
Total study participants	469	251	** **		** **			
**Age groups**	**459**	**243**	** **		** **			
18–29 years	250 (54.5)	148 (60.9)		1			1	
30–59 years	195 (42.5)	87 (35.8)	0.087	1.33	0.96–1.84	0.525	1.23	0.65–2.34
≥60 years	14 (3.1)	8 (3.3)	0.938	1.04	0.42–2.53	0.831	0.88	0.27–2.84
**Gender**	**468**	**250**	** **		** **			
Male	153 (32.7)	82 (32.8)		1			1	
Female	315 (67.3)	168 (67.2)	0.977	1.01	0.73–1.39	0.455	0.76	0.38–1.55
**Living status**	**454**	**241**	** **		** **			
Live without family members (on your own/shared house/others)	86 (18.9)	50 (20.7)		1			1	
Live with family members (partner and/or children)	368 (81.1)	191 (79.3)	0.568	1.12	0.76–1.65	0.125	2.64	0.77–9.10
**Born in Malaysia**	**469**	**251**	** **		** **			
No	48 (10.2)	18 (7.2)		1			1	
Yes	421 (89.8)	233 (92.8)	0.175	0.68	0.39–1.19	0.032	0.33	0.12–0.91
**Completed level of education**	**466**	**250**	*** ***		*** ***			
Secondary	77 (16.5)	36 (14.4)	** **	1	** **		1	
Diploma	83 (17.8)	36 (14.4)	0.792	1.08	0.62–1.88	0.842	1.19	0.21–6.59
Degree (Bachelor)	192 (41.2)	109 (43.6)	0.408	0.82	0.52–1.30	0.899	0.88	0.13–5.90
Masters and above	114 (24.5)	69 (27.6)	0.308	0.77	0.47–1.27	0.083	0.39	0.13–1.13
**Current employment condition**	**460**	**250**	** **		** **			
Unemployed/Home duties	201 (43.7)	108 (43.2)		1			1	
Jobs affected by COVID-19 (lost job/working hours reduced/afraid of job loss)	29 (6.3)	29 (6.3)	0.791	0.92	0.48–1.74	0.103	0.32	0.08–1.26
Have an income source (employed/Government benefits)	230 (50.0)	230 (50.0)	0.944	0.99	0.72–1.36	0.320	0.49	0.12–1.99
**Perceived distress due to change of employment status**	**450**	**249**	** **		** **			
A little to none	315 (70.0)	167 (67.1)	** **	1	** **		1	
Moderate to a great deal	135 (30.0)	82 (32.9)	0.422	0.87	0.63–1.22	NA	NA	NA
**Self-identification as a frontline or essential service worker**	**469**	**251**	** **		** **			
No	327 (69.7)	177 (70.5)		1			1	
Yes	142 (30.3)	74 (29.5)	0.824	1.04	0.74–1.45	0.655	0.89	0.55–1.46
**COVID-19 impacted financial situation**	**469**	**251**	** **		** **			
No	262 (55.9)	117 (46.6)		1			1	
Yes	207 (44.1)	134 (53.4)	***0*.*018***	***0*.*69***	***0*.*51–0*.*94***	0.058	0.73	0.52–1.01
**Co-morbidities**	**395**	**217**						
No	304 (77.0)	174 (80.2)		1			1	
Psychiatric/Mental health problem	11 (2.8)	9 (4.1)	0.437	0.70	0.28–1.72	0.629	1.67	0.21–13.2
Other co-morbidities*	80 (20.3)	34 (15.7)	0.187	1.35	0.87–2.10	0.391	1.34	0.21–13.2
**Smoking**	**469**	**251**	** **		** **			
Never smoker	423 (90.2)	233 (92.8)		1			1	
Ever smoker (Daily/Non-daily/Ex)	46 (9.8)	18 (7.2)	0.236	1.41	0.80–2.48	0.564	1.41	0.44–4.52
**Current alcohol drinking (last 4 weeks)**	**466**	**247**	** **		** **			
No	434 (93.1)	231 (93.5)		1			1	
Yes	32 (6.9)	16 (6.5)	0.844	1.07	0.57–1.98	0.813	1.08	0.56–2.09
**Provided care to a family member/patient with known/suspected case of COVID-19**	**465**	**250**	** **		** **			
No	415 (89.2)	232 (92.8)		1			1	
Yes	50 (10.8)	18 (7.2)	0.123	1.55	0.89–2.73	***0*.*046***	***1*.*87***	***1*.*01–3*.*46***
**Experience related to COVID-19 pandemic**	**442**	**246**	** **		** **			
No known exposure to COVID-19	407 (92.1)	231 (92.1)	** **	1			1	
I had recent overseas travel history and was in self-quarantine	4 (0.9)	6 (2.4)	0.135	0.38	0.11–1.35	NA	NA	NA
I have been tested negative for COVID-19 but self-isolating	31 (7.0)	9 (3.7)	0.084	1.95	0.91–4.18	0.090	3.73	0.81–17.1
**Self-identification as a patient (visited a healthcare provider in the last 4 weeks)**	**468**	**247**	*** ***		*** ***			
No	324 (69.2)	163 (66.0)	*** ***	1	*** ***		1	
Yes	144 (30.8)	84 (34.0)	0.377	0.86	0.62–1.20	0.283	0.83	0.58–1.17
**Healthcare service use in the last 4 weeks**	**184**	**177**	** **		** **			
Telehealth consultation/Use of national helpline	27 (14.7)	18 (15.4)		1			1	
In-person visit to a healthcare provider	144 (78.3)	96 (82.1)	1.000	1.00	0.52–1.92	0.982	1.01	0.51–2.01
Used both services	13 (7.1)	3 (2.6)	0.135	2.89	0.72–11.6	0.133	3.02	0.71–12.8
**Level of psychological distress (K10 categories)**	**469**	**251**	** **		** **			
Low (score 10–15)	198 (42.2)	75 (29.9)		1			1	
Moderate to Very High (score 16–50)	271 (57.8)	176 (70.1)	***0*.*001***	***0*.*58***	***0*.*42–0*.*81***	***0*.*001***	***0*.*54***	***0*.*38–0*.*76***
**Level of fear of COVID-19 (FCV-19S categories)**	**469**	**251**	** **		** **			
Low (score 7–21)	351 (74.8)	174 (69.3)		1			1	
High (score 22–35)	118 (25.2)	77 (30.7)	0.112	0.76	0.54–1.07	0.074	0.72	0.50–1.03
**Healthcare service use to overcome COVID-19 related stress in the last 4 weeks**	**465**	**242**	** **		** **			
No	456 (98.1)	233 (96.3)		1			1	
Yes	9 (1.9)	9 (3.7)	0.153	0.51	0.20–1.31	0.193	0.53	0.21–1.37

Adjusted for: age, gender, living status, born in Malaysia, education and employment

* Cardiac diseases/Stroke/Hypertension/Hyperlipidemia/Diabetes/Cancer/Chronic respiratory disease

## Discussion

This cross-sectional survey found that a large proportion of Malaysian residents experienced moderate to very high levels of psychological distress as a result of the COVID-19 pandemic. Malaysians, whose financial situation was impacted by COVID-19, those who drank alcohol in the past four weeks, those who self-identified as patients and those with higher levels of fear, were more likely to experience higher psychological distress. Higher levels of psychological distress were also associated with higher levels of fear and so were people who self-identified as patients. A large majority of the participants also reported as having medium to highly resilient coping during this pandemic especially those who provided care to family members affected by the pandemic.

Findings of our survey in Malaysia are comparable to similar studies conducted in other parts of the globe. Financial difficulty is associated with anxiety as well as a predisposition to depression after several months of quarantine exacerbated by undue uncertainty [29]. Studies among the general population in China and India have shown that poor economic status and difficulties in meeting living expenses during the COVID-19 pandemic significantly increasing the degree of psychological distress [30, 31]. Likewise, studies during the SARS and MERS epidemics have also shown that increased psychological distress was associated with increased financial difficulties. This could be explained by the emergence of a sense of uncertainty and lack of security during the pandemic [32]. Hence, our finding further supports the inverse association between increased financial difficulties during COVID-19 and the occurrence of psychological distress.

In line with our findings, Ahmed et al. conducted a study in a Chinese population where they had also reported high prevalence of alcohol use and alcohol dependence during the COVID-19 pandemic [33]. Given that this was a cross-sectional study, it was possible that psychological distress led to increased alcohol use as a coping mechanism to deal with COVID-19 induced psychological distress, but the converse was also likely that increased alcohol use worsened psychological distress [34].

This study also showed that people who self-identified as a patient i.e. having visited a healthcare provider in the past four weeks, were more likely to experience higher psychological distress. However, it was not clear from the survey questionnaire if patients had visited a healthcare provider for COVID-19 like symptoms or for other medical conditions. Being infected with COVID-19 or awaiting the possibility of becoming ill was likely to be more stressful because of the fear of mortality or morbidity associated with a disease [29]. Those infected with COVID-19 had higher levels of depression, anxiety, and post-traumatic stress symptoms when compared to those not infected. In fact, people with a history of being infected with COVID-19 had reported unresolved fear, guilt, and helplessness. They were likely to be affected by the stigma of being labelled as someone who had been infected and faced uncertainty about their prognosis and future [35]. Moreover, the findings of this study also highlighted that those who tested negative for COVID-19 but maintained self-isolation from others had higher levels of fear, and those with higher levels of fear of COVID-19 also had moderate to high psychological distress. Knowing the high infectivity capability of the virus, the asymptomatic presentation of some of the COVID-19 positive cases, and the consequences of the COVID-19 infection had created enormous fear among the general population and healthcare workers [36–38]. Unresolved fear which led to long-lasting stress might have predisposed individuals to psychological distress during the COVID-19 pandemic [39]. Hence, our study further strengthened the relationship between fear of the COVID-19 pandemic and increased psychological distress.

Factors identified as protective factors against psychological distress in this study was older age (≥30 years) and having higher level of resilience. Several studies on the psychological impact of COVID-19 in the general population reported that younger people (aged 21 to 40 years) were at higher risk of predisposing to depression and anxiety [40] highlighting consistency with other studies. Younger people may have greater focus on COVID-19 and higher degree of worry about the spread of COVID-19 presumably because of more and/or frequent access to news/social media, hence increasing their risk of psychological distress compared to older people [40].

Those with higher resilience, particularly in the components of tenacity, strength, and optimism, have shown to experience less mental health complications during the COVID-19 pandemic [41]. Our study has also indicated that low resilience was associated with moderate to high levels of psychological distress while moderate to high resilience was not only associated with lower psychological distress, but also enabled the individual to provide care to family members or patients infected with COVID-19. Hence, our study highlighted the pivotal role of resilience in overcoming the psychological impact of the COVID-19 pandemic.

The strength of this study was the use of validated tools to investigate the factors associated with psychological distress, fear and coping strategies in Malaysia. Due to nation-wide travel restrictions, online survey was the only feasible way for data collection, and we were able to recruit a large sample of Malaysian population during the critical pandemic period. However, there were some limitations in this study. As this study was an online survey, most younger people participated into this survey as they were more active on social media. The study was conducted in English, so those who were not well versed in English might not be able to take part in the study. It was beyond the scope of the study to check and ensure that the participants had sufficient ability in understanding English. Due to the self-reporting nature of the survey, possibility of reporting bias cannot be excluded. The survey responses were predominantly from west Malaysia, although the survey link was shared across all the states in Malaysia through various social media platforms and emails. This could be explained by the researchers’ use of snowball sampling techniques which reflected their community acquaintances and accessibility to clinics/allied health service facilities more in West Malaysia than in the eastern part of Malaysia. Another important limitation of our study was, those who might have tested positive to COVID-19 or those whose family members or friends were tested positive with COVID-19 infection or who were interested to this topic were more likely to participate into this survey. We also acknowledge that we might have missed the more marginalized or vulnerable group of population in this study (e.g., those who were more isolated specially people from rural areas, from the areas of poor internet access, older people those who were not active in social media, or migrant or other minority groups); therefore, the findings of this study could be potentially underestimated and might not be representative to the general Malaysian Population.

## Conclusions

The study identified some of the key risk factors for developing psychological distress, fear and coping strategies during the COVID-19 pandemic in Malaysia. Vulnerable groups of individuals such as patients and those impacted financially during COVID-19 should be supported for their mental wellbeing. Behavioural interventions should be targeted to reduce the impact of alcohol drinking during such crisis period. Findings of this study would assist the researchers to plan future studies with vulnerable groups of Malaysians, specifically exploring the strategies to support their mental wellbeing during the pandemic and post-pandemic period. Specific interventions based on the emerging evidence arising from Malaysian and global studies can be tested to alleviate psychological distress, fear and improve resilience among Malaysian population.
