# Supplementary material for: Psychological distress, fear and coping among Malaysians during the COVID-19 pandemic
Source: PLoS One. 2021 Sep 10;16(9):e0257304. doi: 10.1371/journal.pone.0257304 (PMC8432783; doi:10.1371/journal.pone.0257304)
Supplement: Attachment — Submitted filename: PONE-D-20-40506_Response to reviewers_24 Mar 2021.docx [file pone.0257304.s001.docx]

**Response to reviewers**

***Psychological distress, fear and coping among Malaysians during the COVID-19 pandemic (PONE-D-20-40506)***

The line number and page numbers are mentioned according to the track change version of the manuscript

| **Comments from the Editor** | **Responses** |
| --- | --- |
| 1. Please ensure that your manuscript meets PLOS ONE's style requirements, including those for file naming. | The manuscript has been re-formatted accordingly. |
| 2. Please clarify in your Methods section whether the questionnaire is published under a CC-BY license, or whether you obtained permission from the publisher to reproduce the questionnaire in this manuscript. | The questionnaire was not published in the earlier study. Therefore, obtaining permission from the publisher was not applicable. The lead investigator of this study (Rahman MA) was also the lead investigator for the Australian study along with a number of investigators common in both studies. Therefore, no further permission was deemed necessary to use the same study tool for this study. It has already been mentioned: (page-11, line-09)  *“We used the same survey questionnaire (except residence location/region in Malaysia) which was used earlier by the Australian investigators included in this study [7].”* |
| 3. Thank you for stating the following in the Competing Interests section:  "The authors have declared that no competing interests exist."  We note that one or more of the authors are employed by a commercial company: Telstra Health. | This has been addressed and included in the cover letter as advised:  *In terms of funding, I can certify on behalf of all the authors that we did not receive funding for this study. Dr Farhana Sultana is a staff of Telstra Health, but the commercial organization did not fund this study and did not have any role in this study.*  *In addition, I can confirm that we did not have any competing interests. The commercial affiliation does not alter our adherence to PLOS ONE policies on sharing data and materials.*  In addition, we have also updated the manuscript accordingly (Page-19). |
| 4. Thank you for submitting the above manuscript to PLOS ONE. During our internal evaluation of the manuscript, we found significant text overlap between your submission and the following previously published works, some of which you are an author. Please revise the manuscript to rephrase the duplicated text, cite your sources, and provide details as to how the current manuscript advances on previous work. | We have rephrased the entire manuscript, revised and added citations as needed.  In terms of what this study adds further to the existing evidence, we have explained this in the revised Introduction section. |
| **Comments from the Reviewer-1** | **Responses** |
| The study entitled “Psychological distress, fear and coping among Malaysians during the COVID-19 pandemic” investigated the psychological distress, fear and coping strategies as a result of the COVID-19 pandemic among Malaysian residents. Additionally, the factors associated with the psychological distress, fear, and coping strategies are studied and discussed. The sample size is good (n=720) for this study and the analyses seem appropriate (several logistic regression models). However, the presentation and the literature review of the present submission are unsatisfactory. The authors should improve their submission substantially to achieve the scientific rigor. Below please see my specific comments. | We would like to thank the reviewer for the kind feedback. |
| 1. There are many papers on the impacts of COVID-19 on psychological distress. However, the authors did not review these papers properly in their Introduction. I suggest the authors take references from the following publications, which have outlined the psychological distress during COVID-19 in different populations, including children, older people, pregnant women, university students, people with overweight, online population, and general population. I believe that it is very important to enrich the Introduction:   - Nathiya D, Singh P, Suman S, Raj P, Tomar BS. Mental health problems and impact on youth minds during the COVID-19 outbreak: Cross-sectional (RED-COVID) survey. Soc Health Behav 2020;3:83-8 - Lin CY. Social reaction toward the 2019 novel coronavirus (COVID-19). Soc Health Behav 2020;3:1-2 - Chen, C.-Y., Chen, I.-H., Pakpour, A. H., Lin, C.-Y., & Griffiths, M. D. (accepted). Internet-related behaviors and psychological distress among schoolchildren during the COVID-19 school hiatus. Cyberpsychology, Behavior, and Social Networking. - Chen, I.-H., Chen, C.-Y., Pakpour, A. H., Griffiths, M. D., Lin, C.-Y., Li, X.-D., Tsang, H. W. H. (2020). Problematic internet-related behaviors mediate the associations between levels of internet engagement and distress among schoolchildren during COVID-19 lockdown: A longitudinal structural equation modeling study. Journal of Behavioral Addictions. doi: 10.1556/2006.2021.00006 - Pramukti, I., Strong, C., Sitthimongkol, Y., Setiawan, A., Pandin M. G. R., Yen, C.-F., Lin, C.-Y., Griffiths, M. D., Ko, N.-Y. (2020). Anxiety and suicidal thoughts during the COVID-19 pandemic: A cross-country comparison among Indonesian, Taiwanese, and Thai university students. Journal of Medical Internet Research, 22(12), e24487. - Chen, C.-Y., Chen, I.-H., O'Brien, K. S., Latner, J. D., & Lin, C.-Y. (2020). Psychological distress and internet-related behaviors between schoolchildren with and without overweight during the COVID-19 outbreak. International Journal of Obesity. https://doi.org/10.1038/s41366-021-00741-5 - Fazeli, S., Zeidi, I. M., Lin, C.-Y., Namdar, P., Griffiths, M. D., Ahorsu, D. K., Pakpour, A. H. (2020). Depression, anxiety, and stress mediate the associations between internet gaming disorder, insomnia, and quality of life during the COVID-19 outbreak. Addictive Behaviors Reports, 12, 100307. - Mamun, M. A., Sakib, N., Gozal, D., Bhuiyan, A. I., Hossain, S., Bodrud-Doza, M., Mamun, F. A., Hosen, I., Abdullah, A. H., Sarker, M. A., Rayhan, I., Sikder, M. T., Muhit, M., Lin, C.-Y., Griffiths, M. D., Pakpour, A. H. (2021). The COVID-19 pandemic and serious psychological consequences in Bangladesh: a population-based nationwide study. Journal of Affective Disorders, 279, 462-472. - Hashemi, S. G. S., Hosseinnezhad, S., Dini, S., Griffiths, M. D., Lin, C.-Y., Pakpour, A. H. (2020). The mediating effect of the cyberchondria and anxiety sensitivity in the association between problematic internet use, metacognition belief and fear of COVID-19 among Iranian online population. Heliyon, 6(10), e05135. - Ahorsu, D. K., Lin, C.-Y., Pakpour, A. H. (2020). The association between health status and insomnia, mental health, and preventive behaviours: The mediating role of fear of COVID-19. Gerontology and Geriatric Medicine, 6, 1-9. - Lin, C.-Y., Broström, A., Griffiths, M. D., & Pakpour, A. H. (2020). Investigating mediated effects of fear of COVID-19 and COVID-19 misunderstanding in the association between problematic social media use and distress/insomnia. Internet Interventions, 21, 100345. - Chen, I.-H., Chen, C.-Y., Pakpour, A. H., Griffiths, M. D., & Lin, C.-Y. (2020). Internet-related behaviors and psychological distress among schoolchildren during COVID-19 school suspension. Journal of the American Academy of Child and Adolescent Psychiatry, 159(10), 1099-1102. - Ahorsu, D. K., Imani, V., Lin, C.-Y., Timpka, T., Broström, A., Updegraff, J. A., Årestedt, K., Griffiths, M. D., Pakpour, A. H. (accepted). Associations between fear of COVID-19, mental health, and preventive behaviours across pregnant women and husbands: An actor-partner interdependence modelling. International Journal of Mental Health and Addiction. - Chen, C.-Y., Chen, I.-H., Pakpour, A. H., Lin, C.-Y., & Griffiths, M. D. (accepted). Internet-related behaviors and psychological distress among schoolchildren during the COVID-19 school hiatus. Cyberpsychology, Behavior, and Social Networking | We would like to thank the reviewer for this suggestion. In fact, there had been a surge of publications on COVID-19 since we drafted and submitted it to the journal. Based on the feedback from the reviewer and suggested citations, we have carefully reviewed and revised the entire Introduction section by incorporating the relevant references. |
| 2. Given the references I have provided in the comment #1, I think that the statement “However, there has been limited evidence regarding the impact of COVID-19 on community members including healthcare workers” is not supported. Please note that I only provide some references, and there are ample references in the literature that I have not listed. | We do acknowledge the feedback and have updated the sentence with supporting evidence in the Introduction section (Page-9, line-5):  *“There is limited evidence regarding the impact of COVID-19 on psychological distress, fear and coping strategies as a whole and amongst community members and healthcare workers in Malaysia.”* |
| 3. The Methods part provides little information regarding how the authors distribute the online survey. How did the authors use these online platforms to recruit potential participants? | It has been addressed: (page-9, line-21)  *“An online survey link was shared in different online platforms, including Facebook, Twitter and LinkedIn inviting online users to participate in this study.”* |
| 4. How could the authors identify whether the participants were capable of responding to an online questionnaire in English? Specifically, any participant whose English is poor still can answer the English online survey. What the participant has to do is only to click on the answers in random. I wonder how the authors control this factor. Using answering time within 1 minute may work, but not really reflect because the participant whose English is poor still can use a lot of time in answering the online survey. | There was no practical way to control the issue that the reviewer has indicated. We have modified the terms used: (page-10, line-4)  *“To be eligible, participants had to be 18 years or above and were literate enough to respond to an online questionnaire in English.”* |
| 5. The authors said that they used “Snowball sampling technique”, please describe clearly how this technique was used in the online survey. | We have clarified this further: (pgae-10, line-13)  *“Once any participant filled up the online questionnaire, he/she forwarded the survey link to own personal/professional networks.”* |
| 6. After I read the Data Collection subsection, I found that some of my queries above are answered in the subsection. However, this indicates that the authors did not arrange the presentation in a good manner. Therefore, the authors should think about how to clearly deliver the information in their revised manuscript. | We have reviewed and updated the manuscript accordingly. |
| 7. The Study tool subsection is poorly written. The authors should still provide the information on the used instruments. It is quite irresponsive to refer the readers to a previous paper. Specifically, not all readers have time or access to the previous paper. I also wonder why the online survey made no changes given that cultures between Malaysia and Australia are different. | We have updated the section: (page-11, line-9)  *“Three validated tools were included in the survey questionnaire. The Kessler Psychological Distress Scale (K10) tool having ten items was used to assess psychological distress [25], the Fear of COVID-19 scale (FCV-19S) having seven items was used to assess the levels of fear [26], and the Brief Resilient Coping Scale (BRCS) having four items was used to assess the levels of coping [27]. Each of those tools collected responses using a 5-point likert scale and the scoring was categorised as discussed in earlier study [7]. Reliability of using these tools had also been examined in a recent study [28].”*  This study did not have any aim to assess cultural difference or impact of culture on the issues of our interest examined in this study. Therefore, the same questionnaire was used for Australia and Malaysia. |
| 8. Following the previous comment, the authors did not provide proper citations to acknowledge the use of the studied instruments. For example, the citation of Ahorsu et al. (accepted) should be acknowledged for the use of FCV-19S. Similar issues are happened to other used instruments.  Reference:  Ahorsu, D. K., Lin, C.-Y., Imani, V., Saffari, M., Griffiths, M. D., & Pakpour, A. H. (accepted). Fear of COVID-19 Scale: Development and initial validation. International Journal of Mental Health and Addiction | We have addressed this and updated the sub-section of study tool as described above. |
| 9. The authors should provide proper citations to indicate their used cut offs for every studied instruments. | We used the cut-offs as used in previously published studies, which has been mentioned in the sub-section of study tool: (page-11, line-13)  *“Each of those tools collected responses using a 5-point likert scale and the scoring was categorised as discussed in earlier study [7].”* |
| 10. When reporting the significance, please do not use p=0.000. Use p<0.001 instead given that the p-value will never be 0. | We have updated this accordingly. |
