# Supplementary material for: Psychological distress, fear and coping among Malaysians during the COVID-19 pandemic
Source: PLoS One. 2021 Sep 10;16(9):e0257304. doi: 10.1371/journal.pone.0257304 (PMC8432783; doi:10.1371/journal.pone.0257304)
Supplement: Attachment — Submitted filename: PONE-D-20-40506_Response to reviewers.docx [file pone.0257304.s002.docx]

**Response to reviewers**

***Psychological distress, fear and coping among Malaysians during the COVID-19 pandemic (PONE-D-20-40506)***

The line number and page numbers are mentioned according to the track change version of the manuscript

| **Comments from the Reviewer-1** | **Responses** |
| --- | --- |
| I would like to thank the reviewers that they have sincerely take my prior comments into consideration to prepare this revision. I can observe that the revised manuscript is substantially improved. I have only one minor comment for the authors to further address. That is, the authors acknowledged that they were unable to make sure that all the participants having sufficient ability in understanding English. Then, this should be listed as one of the limitations. | We would like to thank the reviewer for the kind feedback.  The minor comments have been addressed and we have clarified this in the limitation section (page-16, line-17):  *“The study was conducted in English, so those who were not well versed in English might not be able to take part in the study. It was beyond the scope of the study to check and ensure that the participants had sufficient ability in understanding English.”* |
