# Supplementary material for: Psychological distress, fear and coping among Malaysians during the COVID-19 pandemic
Source: PLoS One. 2021 Sep 10;16(9):e0257304. doi: 10.1371/journal.pone.0257304 (PMC8432783; doi:10.1371/journal.pone.0257304)
Supplement: Attachment — Submitted filename: Reviewer 2 Comments.docx [file pone.0257304.s003.docx]

Reviewer #2: Manuscript ID: PONE-D-20-40506R2
1. Recommendation
Minor revision

2. Comments to Author:
Thank you for the opportunity of review this study entitled “Psychological distress, fear and coping among Malaysians during the COVID-19 pandemic”. The manuscript presented an investigation about the levels of psychological distress, fear and coping strategies and other related factors among Malaysian residents. The framework of this work is the COVID-19 pandemic.
In my opinion, the study has some relevant and interesting results. I think it's a good paper, well done. I commend the authors and other reviewers who have worked to reach this optimum point. There are only a few very minor issues that need to be addressed before the document is suitable for publication.
• Authors should be more detailed in the limitation section. The use of the online survey implies the need to pay attention in generalize results to general population, since people who do not have internet access (pronominally older individuals, of course) could be unrepresented. Furthermore, participants were voluntary, so only those who have interest in this research topic decided to participate: they may be unrepresentative of general Malaysian population.
• There are no incentives for future research: it is important not only to find a link with past results, but also to offer possible ideas for future work, in order to favour a continuous development of research. Therefore, please add implications for future research
• Parallelly, the paper could benefit from a focus on its strengths, highlighting the relevance of this research topic and results.
• In the conclusion section, the authors should focus on the practical implications of this study. Some studies provided an understanding of the treat responses at the time of COVID-19, as well as the effect of stress on health. Given these outcomes and the large incidence of these variables highlighted in Malaysian residents, the importance of effective tailor-made interventions on protective/risk variables (e.g., coping, defences etc.. based on previous studies) could be highlighted. I think the authors have a wide choice, given the large amount of literature available on this field. Therefore, the results of this study highlight the importance of working on some outcames (e.g., distress and fear) related to COVID-19 in Malaysian residents, preparing interventions that work on other associated variables, both those included in the study and other explored in previous research.
