# Supplementary material for: Psychological distress, fear and coping among Malaysians during the COVID-19 pandemic
Source: PLoS One. 2021 Sep 10;16(9):e0257304. doi: 10.1371/journal.pone.0257304 (PMC8432783; doi:10.1371/journal.pone.0257304)
Supplement: Attachment — Submitted filename: PONE-D-20-40506R2_Response to reviewers_06 Aug 2021.docx [file pone.0257304.s004.docx]

**Response to reviewers**

***Psychological distress, fear and coping among Malaysians during the COVID-19 pandemic (PONE-D-20-40506R2)***

The line number and page numbers are mentioned according to the track change version of the manuscript

| **Comments from the Reviewer-2** | **Responses** |
| --- | --- |
| Thank you for the opportunity of review this study entitled “Psychological distress, fear and coping among Malaysians during the COVID-19 pandemic”. The manuscript presented an investigation about the levels of psychological distress, fear and coping strategies and other related factors among Malaysian residents. The framework of this work is the COVID-19 pandemic.  In my opinion, the study has some relevant and interesting results. I think it's a good paper, well done. I commend the authors and other reviewers who have worked to reach this optimum point. There are only a few very minor issues that need to be addressed before the document is suitable for publication. | We would like to thank the reviewer for the kind feedback.  The minor comments have been addressed in the revised version. |
| Authors should be more detailed in the limitation section. The use of the online survey implies the need to pay attention in generalize results to general population, since people who do not have internet access (pronominally older individuals, of course) could be unrepresented. Furthermore, participants were voluntary, so only those who have interest in this research topic decided to participate: they may be unrepresentative of general Malaysian population. | We have addressed this and expanded the limitation section further:  (Page:16, Line: 22)  *Due to the self-reporting nature of the survey, possibility of reporting bias cannot be excluded.*  (Page: 17, Line: 04)  *Another important limitation of our study was, those who might have tested positive to COVID-19 or those whose family members or friends were tested positive with COVID-19 infection or who were interested to this topic were more likely to participate into this survey. We also acknowledge that we might have missed the more marginalized or vulnerable group of population in this study (e.g., those who were more isolated specially people from rural areas, from the areas of poor internet access, older people those who were not active in social media, or migrant or other minority groups); therefore, the findings of this study could be potentially underestimated and might not be representative to the general Malaysian Population.* |
| There are no incentives for future research: it is important not only to find a link with past results, but also to offer possible ideas for future work, in order to favour a continuous development of research. Therefore, please add implications for future research | We have added future research areas (Page: 17, Line: 21)  *Findings of this study would assist the researchers to plan future studies with vulnerable groups of Malaysians, specifically exploring the strategies to support their mental wellbeing during the pandemic and post-pandemic period.* |
| Parallelly, the paper could benefit from a focus on its strengths, highlighting the relevance of this research topic and results. | We have added strengths (Page:16, Line: 13)  *The strength of this study was the use of validated tools to investigate the factors associated with psychological distress, fear and coping strategies in Malaysia. Due to nation-wide travel restrictions, online survey was the only feasible way for data collection, and we were able to recruit a large sample of Malaysian population during the critical pandemic period.* |
| In the conclusion section, the authors should focus on the practical implications of this study. Some studies provided an understanding of the treat responses at the time of COVID-19, as well as the effect of stress on health. Given these outcomes and the large incidence of these variables highlighted in Malaysian residents, the importance of effective tailor-made interventions on protective/risk variables (e.g., coping, defences etc.. based on previous studies) could be highlighted. I think the authors have a wide choice, given the large amount of literature available on this field. Therefore, the results of this study highlight the importance of working on some outcames (e.g., distress and fear) related to COVID-19 in Malaysian residents, preparing interventions that work on other associated variables, both those included in the study and other explored in previous research | We have already included implications of current findings (Page:17, Line:18)  *Vulnerable groups of individuals such as patients and those impacted financially during COVID-19 should be supported for their mental wellbeing. Behavioural interventions should be targeted to reduce the impact of alcohol drinking during such crisis period.*  We have also added another implication (Page: 17, Line: 24)  *Specific interventions based on the emerging evidence arising from Malaysian and global studies can be tested to alleviate psychological distress, fear and improve resilience among Malaysian population.* |
